# Deficiency of Insulin-Like Growth Factor-1 Receptor Confers Resistance to Oxidative Stress in C2C12 Myoblasts

**DOI:** 10.1371/journal.pone.0063838

**Published:** 2013-05-10

**Authors:** Sachin Thakur, Neha Garg, Martin L. Adamo

**Affiliations:** 1 Department of Biochemistry, The University of Texas Health Science Center at San Antonio, San Antonio, Texas, United States of America; 2 The Sam and Ann Barshop Institute for Longevity and Aging Studies at The University of Texas Health Science Center at San Antonio, San Antonio, Texas, United States of America; University of Illinois at Chicago, United States of America

## Abstract

IGF-1 receptor (IGF-1R) signaling regulates cell growth, transformation and survival. Haploinsufficiency of the IGF-1R is reported to paradoxically confer resistance to oxidative stress in vivo and in cells cultured from *Igf1r^+/−^* mice. In order to determine whether IGF-1R deficiency directly confers resistance to oxidative stress in specific cell types, an siRNA-mediated approach was applied to reduce IGF-1R in C2C12 myoblasts, NIH3T3 fibroblasts and MC3T3-E1 osteoblasts. Treating the IGF-1R deficient myoblasts with H_2_O_2_ resulted in significantly higher phosphorylation of Akt as compared to cells having normal expression of IGF-1R. Similar results were obtained with UV treatment, another inducer of oxidative stress. This enhanced activation of Akt was associated with reduced level of cleaved caspase-3 and PARP. Moreover, in the IGF-1R knockdown myoblasts, phosphorylation of the Akt substrate Bad was enhanced after peroxide treatment. However, in NIH-3T3 fibroblasts and MC3T3-E1 osteoblasts, the loss of IGF-1R by siRNA directed knockdown was associated with reduced levels of phosphorylated Akt on treatment with H_2_O_2_ or UV as compared to control cells and these cells showed more apoptosis. These results suggest a novel mechanism of cell type specific differential regulation of resistance to oxidative stress induced apoptosis by reduced levels of IGF-1R.

## Introduction

The antiapoptotic effect of the IGF-1/IGF-1 receptor (IGF-1R) signaling is a well established pathway for cell survival in a wide array of cell types, and is mainly mediated through PI3 kinase/Akt and Ras–Raf–MAPK pathways [1], [2]. Moreover IGF-1R signaling is important in the formation, progression and metastatic spread of many cancer types and provides resistance to anti-cancer drugs [3]. Apoptosis is one of the mechanisms to slow the progression of tumors; blocking survival signaling mediated by IGF-1/IGF-1R has been one of the approaches in the development of anticancer therapies [4], [5]. However, there is evidence that IGF-1R may be intrinsically pro-apoptotic [6]. Overexpression of IGF-1R can induce apoptosis in cultured cells [7], [8] and deficiency of the IGF-1R can cause cell proliferation and hyperplasia [9].

Genetic alterations in the evolutionarily conserved insulin/IGF-1 signaling pathway lead to life span extension in various animal models ranging from *Caenorhabditis elegans* to mice [10]. In 2003, Holzenberger et al. [11] reported that female mice haploinsufficient for the IGF-1R (*Igf1r^+/−^*) live longer and show resistance to oxidative stress. Moreover, embryonic fibroblasts isolated from these mutants were more resistant to oxidative stress induced by hydrogen peroxide. In another study done on *Igf1r* ^−/−^ mouse embryonic fibroblasts (MEFs), absence of IGF-1R reduces DNA-damage–induced apoptosis through reduced translational synthesis of p53 and Mdm2 expression [12]. These findings suggest that reduction in IGF-1R can activate alternative pathways for averting stress-induced apoptosis in addition to primary pathways. There is limited knowledge of the mechanism of this phenomenon; hence there is a need to assess the phenotype of oxidative stress resistance mediated by reduced IGF-1R activation. In the present study, we used three cell lines, C2C12 myoblasts, NIH3T3 fibroblasts, and MC3T3-E1 osteoblasts to investigate oxidative stress resistance associated with acute IGF-1R deficiency generated by RNA silencing. IGF-1R deficiency conferred resistance to oxidative stress only in C2C12 myoblasts, as compared to myoblasts having normal expression of IGF-1R, an effect associated with increased Akt phosphorylation and reduced levels of apoptotic markers. In NIH3T3 fibroblasts and MC3T3-E1 osteoblasts, RNA silencing of IGF-1R led to reduced Akt phosphorylation and increased apoptosis. Our results thus support the notion that reductions in IGF-1R paradoxically confer resistance to oxidative stress in a cell specific manner.

## Materials and Methods

### Materials

NIH3T3 fibroblasts and MC3T3-E1 osteoblasts were purchased from American Type Culture Collection (ATCC) (Manassas,VA), and C2C12 murine myoblasts originally purchased from ATCC were provided by Dr. John C. Lee (University of Texas Health Science Center at San Antonio). Wortmannin, H_2_O_2_, 4′,6-Diamidino-2-phenylindole (DAPI) and bovine serum albumin (fraction V) were purchased from Sigma-Aldrich Co. (St. Loius, MO). Recombinant human IGF-I (rhIGF-I) was purchased from Austral Biologicals (San Ramon, CA). All antibodies were obtained from Cell Signaling Technologies (Beverly, MA), and secondary anti-rabbit antibody was obtained from Santa Cruz (Santa Cruz, CA).

### Cell Culture Conditions and siRNA Transfections

C2C12 myoblasts and NIH3T3 fibroblasts were maintained in high-glucose Dulbecco’s modified Eagle’s medium (DMEM) containing 10% heat inactivated fetal bovine serum (FBS) and penicillin (100 U/ml) and streptomycin (100 µg/ml), at 37°C in a humidified atmosphere with 5% CO2. MC3T3-E1 osteoblasts were maintained in alpha MEM. For RNA silencing of the IGF-1R in cells, pre-designed Silencer Select siRNAs for mouse IGF-1R (ID# s68115) and negative control (non-targeting siRNA ID# 4390843) were purchased from Ambion Inc. (Austin, TX). Cells (1.5×10^5^/well in 6-well culture plate for C2C12 and NIH3T3, and 1.0×10^5^/well for MC3T3-E1) were reverse-transfected with double-stranded siRNA in antibiotic-free media plus 10% FBS using Lipofectamine 2000 (Invitrogen, Carlsbad, CA) according to manufacturer’s instructions. After 24 h of reverse transfection, medium was changed to normal medium containing antibiotics. Cells were either treated after 24 h at 90–95% confluence, or as otherwise stated.

### RNA Isolation, cDNA Synthesis, and Real-time PCR

Cells were harvested at 90–95% confluence for total RNA isolation using RNA STAT-60 (Tel-test, Friendswood, TX, USA). Double stranded cDNA was synthesized from 2.0 µg of RNA using the High-capacity cDNA Archive Kit (P/N 4322171; ABI, Foster City, CA, USA) following manufacturer’s protocol. cDNA samples were stored at −80°C until use. TaqMan Universal PCR Master Mix (P/N 4324018) and TaqMan-MGB probes for IGF-1R (Mm00802831_m1) and B2M (beta-2-microglobulin) (Mm00437762_m1) were purchased from ABI. The real-time PCR reactions were performed within an ABI 7500 thermal cycler. Data were collected and analyzed by ABI 7500 Fast System SDS (v 1.3.0) software. All samples were run in duplicate.

### UV Irradiation

For standardizing dose of UV irradiation, cells (C2C12 myoblasts, NIH3T3 fibroblasts and MC3T3-E1 osteoblasts) were seeded in 35 mm plates (2 ml medium) and grown to 90–95% confluence. Cells were exposed to UV irradiation (100 J/m^2^ and 500 J/m^2^) by using UV Crosslinker FB-UVXL-1000 (Fisher Scientific, Pittsburgh, PA). Lids were removed during exposure. Cells were further incubated post-exposure for 30 min. A UV dose of 200 J/m^2^ was finally used for the experiment.

### Protein Extraction and Western Blot

After removing the media, cells were washed thrice with ice-cold PBS and lysed in ice-cold lysis buffer containing 20 mM Tris-HCl (pH 7.5), 150 mM NaCl, 1 mM Na_2_EDTA, 1 mM EGTA, 1% Triton, 2.5 mM sodium pyrophosphate, 1 mM beta-glycerophosphate, 1 mM Na_3_VO_4_, 1 µg/ml Leupeptin, 1 mM PMSF, and 1∶100 dilution of phosphatase inhibitor cocktails 1 and 2 (Sigma). Lysates were then centrifuged at 12000 rpm for 20 min at 4°C. Proteins were quantified. For Western blotting, equal amounts of cell lysate proteins (typically 25 µg) were separated by denaturing SDS-PAGE and transferred electrophoretically to PVDF membranes (Millipore Corp., Bedford MA). Membranes were incubated for 1 h in 5% dry milk solution in TBST (20 mM Tris-HCl, pH 7.5, 0.5 M NaCl, 1% Tween 20) for blocking. After washing thrice with TBST, membranes were incubated with primary antibody in 5% BSA in TBST overnight at 4°C. Membranes were washed three times in TBST followed by incubation with secondary antibody for 1 h at room temperature and again washed three times at the end of incubation. Proteins were detected by incubating the PVDF membrane with enhanced chemiluminescence reagents (Thermo Fisher, Rockford, IL) followed by exposing to X-ray film.

### DAPI Staining

C2C12 myoblasts were reverse-transfected in the same manner as mentioned above, except that they were grown in 35 mm plate having coverslip placed inside. After reaching 70–80% confluence, cells were treated with H_2_O_2_ (400 µM) for 10 hours. Cells were stained with DAPI (1 µg/ml) for 10 min and washed twice with PBS. Cells were fixed for 20 min at room temperature with 4% paraformaldehyde, and washed thrice with PBS. Coverslips were mounted on glass slides using ProLong® Gold antifade reagent (Invitrogen, Carlsbad, CA). For quantitation of cell nuclei and apoptotic nuclei, cells were visualized using an Olympus JP/1X71 (Melville, NY) fluorescence microscope with digital camera output.

### Statistics

Data are presented as means ± S.E.M. Statistics was performed using Student’s t-test. P-value <0.05 was considered significant.

## Results

### Effect of IGF-1R Knockdown on Cell Proliferation

**Figure 1:**
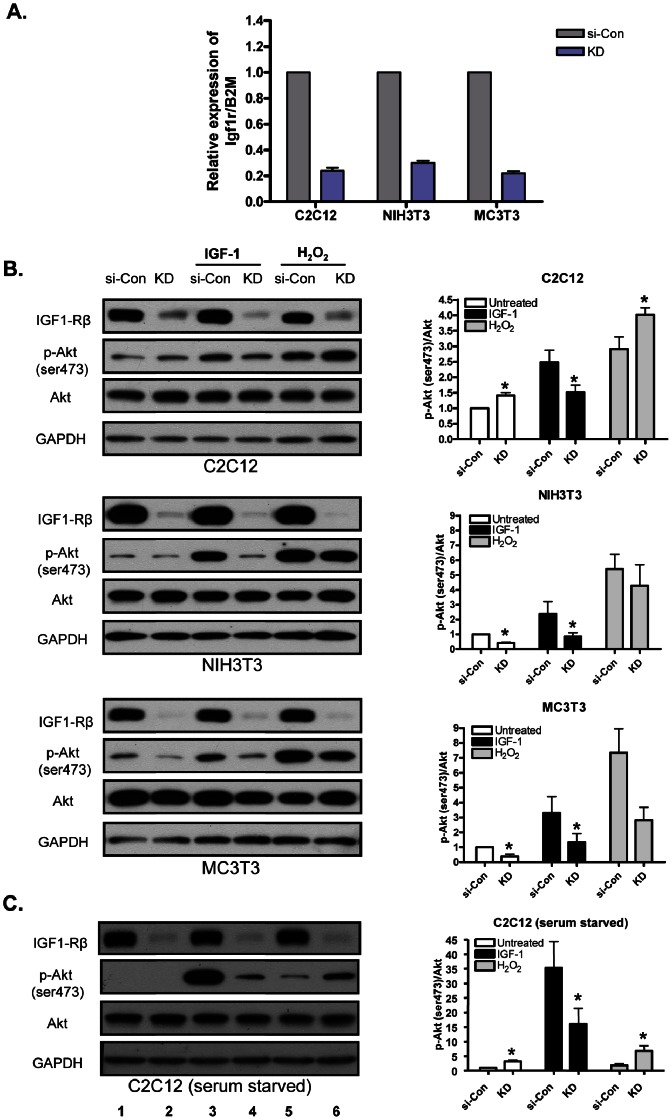
IGF-1R deficiency in C2C12 myoblasts leads to increase in Akt phosphorylation on H_2_O_2_ treatment. (A) siRNA mediated knockdown efficacy of *Igf1r* gene was determined by real-time PCR using B2M as reference gene. (B) Cells were reverse-transfected in 6-well plates with 10 nM negative control siRNA (si-Con) and 10 nM siRNA against IGF1R (KD). Medium was changed after 24 h, and cells were maintained for another 24 h (90–95% confluent). After 48 h of initial transfection, cells were treated with rhIGF-1 (125 ng/ml) for 30 min (lane 3 and 4), H_2_O_2_ (400 µM) for 30 min (lane 5 and 6). Then cells were harvested for protein lysates and subjected to Western blot analysis. (C) C2C12 myoblasts were serum starved for 2 h (0.1% BSA in DMEM) and treated in same manner as described in (B). si-Con represents cells transfected with non targeting siRNA as negative control and KD represents cells transfected with siRNA against IGF-1R. Right panel represents densitometric analysis of the ratio of p-Akt and total Akt bands. Values are the means ±S.E. from three independent experiments. *, denotes significant differences between KD cells and si-Con cells (*p*<0.05). p values were determined by Student’s *t* test.

We first determined efficacy of siRNA-mediated knockdown of IGF-1R. Cells (C2C12, NIH3T3 and MC3T3-E1) were reverse transfected with 10 nM siRNA against IGF-1R (KD) or non-targeting control siRNA (si-Con). si-IGF-1R transfection reduced *Igf1r* expression by ∼70–80% (Figure 1A). To determine the IGF-1R knockdown effect on cell proliferation, cells were counted at two time points, 24 and 48 h after transfection. There was no significant reduction in cell numbers at any time point (data not shown).

### IGF-1R Deficiency in C2C12 Myoblasts Leads to Increase in Akt Phosphorylation on H_2_O_2_ Treatment

To investigate the effect of IGF-1R deficiency on H_2_O_2_ induced Akt phosphorylation, cells at 90–95% confluence were treated with H_2_O_2_ (400 µM) for 30 min. Interestingly, we found that on H_2_O_2_ treatment, there was significantly higher Akt phosphorylation in IGF-1R deficient (KD) C2C12 myoblasts as compared to control (si-Con) C2C12 myoblasts. Conversely, NIH3T3 and MC3T3-E1 cells showed reduced Akt phosphorylation in KD cells as compared to their respective si-Con cells. IGF-1R knockdown also caused a significant reduction in basal phospho-Akt levels in NIH3T3 and MC3T3-E1 cells. On the contrary, KD-C2C12 myoblasts showed significantly higher basal phospho-Akt levels (Figure 1B). To exclude confounds produced by growth factors present in serum, C2C12 cells at 90–95% confluence were serum starved for 2 h and treated in same manner as described above. Similar results were obtained with serum-starved cells as were observed with complete growth medium (serum starved data shown only for C2C12 myoblasts, Figure 1C).

The action of PI3K is negatively regulated by PTEN (phosphatase and tensin homologue deleted on chromosome 10), a tumor suppressor gene encoding a phosphatase, which dephosphorylates phosphatidylinositol (3,4,5)-trisphosphate (PtdIns (3,4,5)*P*_3_ or PIP_3_) to PIP_2_ (PtdIns(4,5)P2) [13]. It has been shown that H_2_O_2_ activates PI3Ksignaling via the inactivation of PTEN [14], [15]. To determine whether H_2_O_2_ treatment affects IGF-1 induced signaling via a PTEN mechanism, we pretreated C2C12 myoblasts with IGF-1 followed by H_2_O_2_ treatment and vice versa (pretreatment with H_2_O_2_ followed by IGF-1). No difference was observed with pre-or post treatment of H_2_O_2_ - KD-C2C12 myoblasts still showed reduced phospho-Akt level as compared to si-Con-C2C12 myoblasts (Figure S1). This result indicates that IGF-1 dependent signaling was not affected by H_2_O_2_.

The IGF1R mediates its anti apoptotic effects via activation of the phosphatidylinositol 3-kinase (PI3K), leading to phosphorylation of Akt, which catalyzes inhibitory phosphorylation of the proapoptotic molecule Bad [16]. In order to confirm whether H_2_O_2_ induced Akt phosphorylation in KD-C2C12 myoblasts is PI3K mediated, we treated the C2C12 myoblasts with wortmannin (pharmacological inhibitor of PI3K) prior to H_2_O_2_ treatment. Wortmannin effectively suppressed the IGF-1 and H_2_O_2_ induced Akt phosphorylation in both si-Con and KD-C2C12 myoblasts (Figure S2A). These results indicate that deficiency of IGF-1R in C2C12 myoblasts results in significantly higher Akt phosphorylation, which is PI3K mediated.

### IGF-1R Deficiency in C2C12 Myoblasts Leads to Increase in Akt Phosphorylation on UV Treatment

**Figure 2:**
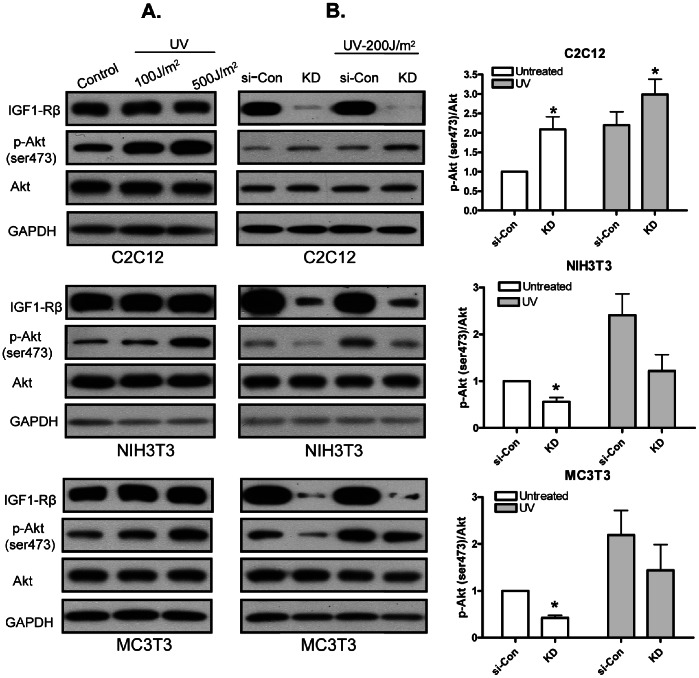
IGF-1R deficiency in C2C12 myoblasts leads to increase in Akt phosphorylation on UV treatment. (A) Cells were grown in 35 mm plate. After reaching 90–95% confluence, cells were exposed to UV treatment of 100 J/m^2^ and 500 J/m^2^. Plates were again incubated for 30 min. Cells were harvested for protein lysates and subjected to Western blot analysis. (B) Cells were reverse transfected and maintained as described in Figure 1B. Cells were exposed to 200 J/m^2^ of UV irradiation. Plates were re-incubated for 30 min. Cells were harvested for protein lysates and subjected to Western blot analysis. Right panel represents densitometric analysis of the ratio of p-Akt and total Akt bands. Values are the means ±S.E. from three independent experiments. *, denotes significant differences between KD cells and si-Con cells (*p*<0.05). p values were determined by Student’s *t* test.

We used UV radiation as another stressor for producing endogenous H_2_O_2_ which induces Akt phosphorylation [17]. To standardize the dose/intensity of UV radiation, cells were exposed to two different UV intensities, 100 J/m^2^ and 500 J/m^2^. Both the doses markedly enhanced Akt phosphorylation (Figure 2A). Finally, cells were exposed to 200 J/m^2^ of UV radiation. Results obtained for Akt phosphorylation were similar to those obtained with exogenous H_2_O_2_ treatment. There was significant increase in Akt phosphorylation in KD-C2C12 myoblasts as compared to si-Con-C2C12 myoblasts, whereas KD-NIH3T3 and KD-MC3T3-E1 cells showed reduced Akt phosphorylation compared to their respective control cells (Figure 2B). These results further strengthen our conclusion that reduced levels of the IGF-1R in C2C12 myoblasts result in significantly higher Akt phosphorylation.

### IGF-1R Deficiency Protects C2C12 Myoblasts from H_2_O_2_-induced Apoptosis

**Figure 3:**
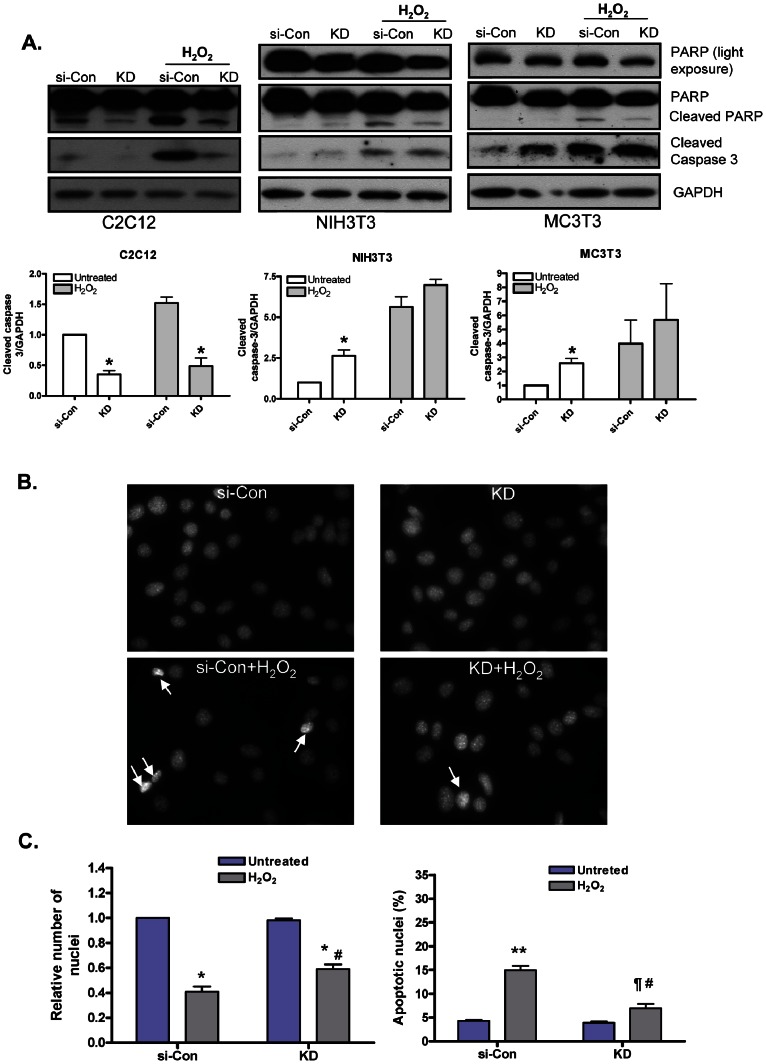
IGF-1R deficiency protects C2C12 myoblasts from H_2_O_2_-induced apoptosis. (A) Cells were reverse transfected and maintained as described in Figure 1B. Cells were treated with H_2_O_2_ (400 µM) for 4-hours and then harvested for protein lysates. Western blots were performed to detect levels of Caspase-3 and PARP, (cleaved Caspase-3-17 kDa, and cleaved PARP –85 kDa). Lower panel, represents densitometric analysis of the ratio of cleaved caspase 3 and GAPDH bands. Values are the means ±S.E. from three independent experiments. *, denotes significant differences between KD cells and si-Con cells (*p*<0.05). p values were determined by Student’s *t* test. (B) Total nuclei and apoptotic nuclei of C2C12 myoblasts were visualized by DAPI staining. Apoptotic nuclei were identified by their typical nuclear appearance; condensed and deformed nuclei as bright spots (white arrows). (C) Relative total nuclei and percentage of apoptotic nuclei were scored by counting nuclei in at least eight fields (n≥125 each field). Data shown are average of three independent experiments. Error bar indicates SEM. *, indicates significant difference from si-Con myoblasts (Untreated) (p<0.01). **, indicate significant difference between si-Con myoblasts (Untreated and H_2_O_2_ treated) (p<0.01). ¶, indicates significant difference between KD myoblasts (Untreated and H_2_O_2_ treated) and #, indicates significant difference between si-Con (H_2_O_2_ treated) and KD myoblasts (H_2_O_2_ treated) (p<0.01). p values were determined by Student’s *t* test.

The role of Akt in driving anti-apoptotic signaling pathways in cells is well documented [18]. To determine whether significantly higher activation of Akt on H_2_O_2_ treatment, protects KD-C2C12 myoblasts from apoptosis, biochemical markers of apoptosis including Caspase-3 and Poly (ADP)-ribosyl-polymerase (PARP) were determined. Cells at 90–95% confluence were treated with H_2_O_2_ (400 µM) for 4 h. In KD-C2C12 myoblasts, both basal and H_2_O_2_-induced levels of cleaved Caspase-3 and PARP were significantly less as compared to si-Con-C2C12 myoblasts (Figure 3A), which was in accordance with higher level of phospho-Akt in KD-C2C12 myoblasts. Conversely, KD-NIH3T3 and KD-MC3T3-E1 cells exhibited greater cleavage of Caspase-3 as compared to their respective si-Con cells (Figure 3A). In NIH3T3 and MC3T3-E1 cells, knocking down of IGF-1R reduced total PARP expression (116 kDa, upper band) (Figure 3A). Hence, interpretation of PARP cleavage in these cell lines was difficult. Moreover, pretreatment with wortmannin resulted in increased cleavage of H_2_O_2_-induced caspase 3 and PARP levels in both si-Con and KD-C2C12 myoblasts (Figure S2B), albeit the cleavage was more pronounced in the si-Con-C2C12 myoblasts. To further validate our findings in C2C12 myoblasts, we performed DAPI staining. We found that treating cells with H_2_O_2_ (400 µM) for 10 h (to observe greater effect of treatment) resulted in a significant reduction in si-Con-C2C12 nuclei number by ∼59% (p<0.01) and KD-C2C12 nuclei number by ∼41% (p<0.01). However, the reduction in total nuclei numbers (H_2_O_2_ treated) was significantly less for KD-C2C12 myoblasts when compared to si-Con-C2C12 myoblasts (p<0.01). In addition, the apoptotic nuclei counted in si-Con-C2C12 (H_2_O_2_treated) myoblasts were ∼15% of total nuclei (sum of intact and apoptotic nuclei), which was significantly higher than the KD-C2C12 myoblasts, which showed ∼7% apoptotic nuclei (p<0.01). These results suggest that deficiency of IGF-1R in C2C12 myoblasts confers resistance to oxidative stress by enhancing Akt phosphorylation.

### Effect of Akt Activation on its Downstream Substrate Bad

**Figure 4:**
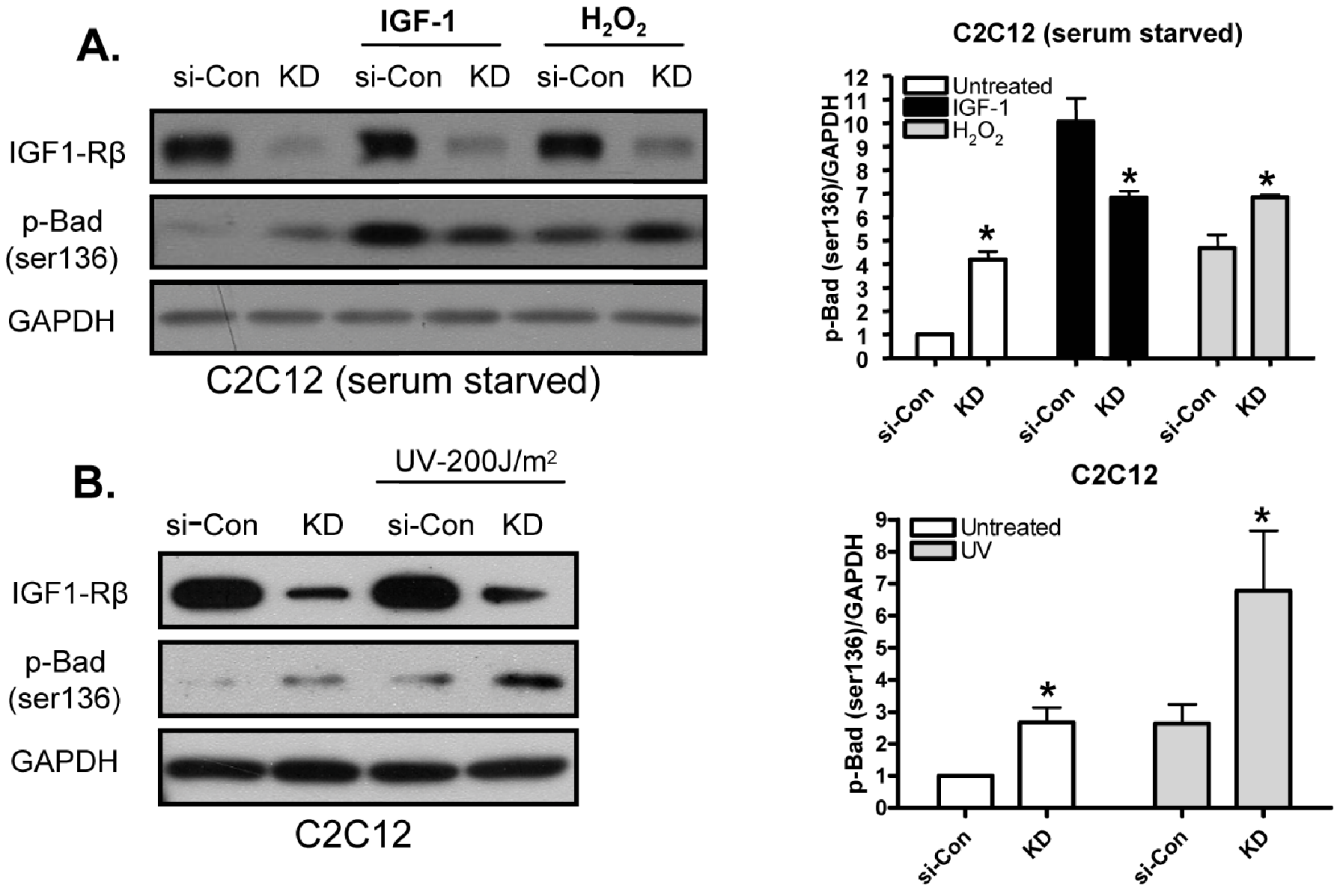
Effect of Akt activation on its downstream substrate Bad in C2C12 myoblasts. (A) Cell lysates from C2C12 myoblasts experiments (30 min signaling, serum starved) were used for analyzing p-Bad (ser136) by Western blot. (B) Cell lysates from C2C12 myoblasts UV experiments were used for analyzing p-Bad (ser136) by Western blot. Right panel represents densitometric analysis of the ratio of p-Akt and total Akt bands. Values are the means ±S.E. from three independent experiments. *, denotes significant differences between KD cells and si-Con cells (*p*<0.05). p values were determined by Student’s *t* test.

Bad promotes apoptosis through the mitochondrial intrinsic death pathway. Akt phosphorylates Bad at serine 136, resulting in dissociation of Bad from Bcl-2/Bcl-XL and increased Bad association with cytosolic 14-3-3, thereby suppressing pro-death signals (Datta et.al., Cell, 1997). Our result demonstrate that p-Bad (ser136) was significantly higher in basal as well as H_2_O_2_ (Figure 4A) or UV (Figure 4B) treated KD-C2C12 myoblasts. Our data with p-Bad (ser136) is consistent with increased Akt activation in oxidatively stressed C2C12 cells deficient in IGF-1R, suggesting that Akt exerts its anti apoptotic effect through its downstream substrate Bad.

## Discussion

IGF-1R is a membrane-spanning tyrosine kinase receptor which plays an important role in survival and antiapoptotic pathways through its downstream regulator Akt. On activation, Akt inactivates various pro-apoptotic proteins like, Ask1 [19], BAD [16], Bax [20], FoxO transcription factors [21] and caspase-9 [22] by phosphorylating them on specific sites. In the present study we found that deficiency of IGF-1R in C2C12 myoblasts paradoxically confers resistance to oxidative stress in association with significantly enhanced Akt phosphorylation as compared to control C2C12 myoblasts. The results of Caspase-3 and PARP cleavage assays were in accordance with Akt phosphorylation; more phosphorylation of Akt was associated with reduced cleavage of caspase-3 and PARP, reflecting decreased apoptosis. Moreover, phosphorylation of Bad at the Ser136 Akt phosphorylation site was enhanced in KD-C2C12 cells treated with H_2_O_2_.

Our findings in C2C12 myoblasts deficient in IGF-1R are similar to Holzenberger *et al.* [11], where they showed that MEFs isolated from *Igf1r^+/−^* mice were more resistant to oxidative stress induced by hydrogen peroxide. Resistance to oxidative stress was explained by hypophosphorylation of p66 Shc in IGF-1R deficient MEFs. However, the role of Akt in mediating resistance to oxidative stress was not demonstrated. Loss of IGF-1R activity in MEFs isolated from *Igf1r* ^−/−^ mice led to reduced DNA-damage–induced apoptosis due to decreased expression of p53 and Mdm2 [12]. The observed phenotype was independent of the Akt pathway since absence of IGF-1R caused inactivation of PI3K-Akt pathway. In our study, we found that pre-treatment of KD-C2C12 myoblasts and si-Con-C2C12 myoblasts with wortmannin effectively inhibited H_2_O_2_ induced Akt phosphorylation suggesting PI3K dependent Akt activation by H_2_O_2_. Additionally, wortmannin pretreatment increased the levels of H_2_O_2-_induced cleaved Caspase-3 and PARP in both si-Con- and KD- C2C12 myoblasts. However, cell protection effect conferred by IGFIR deficiency in C2C12 myoblasts was only partially abolished.

Recently, it has been reported that brown preadipocytes, in which both insulin and IGF-1 receptors have been knocked out, were resistant to apoptosis when compared to wild type cells or cells lacking either receptor alone [23]. The authors suggested that in the absence of ligand, these receptors (IR and IGF-1R) might generate proapoptotic signals. It has been shown previously that growth factor receptors have the potential to sensitize cells to apoptosis despite being modulators of cell survival pathways [6], [24], [25]. Another potential reason for resistance to apoptosis may be that reduced IGF-1R signaling, resulting from genetic manipulations or use of pharmacological inhibitors, can be compensated for by increased signaling through other receptors to effect cell survival [26], [27]. To determine the possibility of compensatory increase in endogenous IGF-1 levels, in turn activating the IGF-1R and thereby leading to higher Akt phosphorylation (basal or H_2_O_2_ induced), we also determined *Igf1* expression in KD-C2C12 myoblasts. However, no change was observed in the *Igf1* mRNA levels (data not shown).

Hydrogen peroxide is a strong oxidant that can cause apoptosis, at least in part, through a mechanism involving the intrinsic mitochondrial death pathway [28], [29]. H_2_O_2_ alone can induce activation of downstream effectors PI3K-Akt and MAPK via tyrosine kinase receptor dependent [30] or independent pathways [31], [32]. H_2_O_2_ is also known to activate PI3K signaling via the inactivation of PTEN [14], [15]. However, in our study IGF-1 dependent signaling was not affected by H_2_O_2_. Based on evidence from literature and our current findings, it is conceivable that various mechanisms are involved in providing resistance to oxidative stress in cells having disrupted IGF-1R signaling. Distrupting IGF-1R signaling alone may not be effective in inhibiting progression of some cancer types [9], [33]. The level of expression of functional IGF-1R may be a critical determinant in deciding its role as antiapoptotic or proapoptotic *in vitro* and *in vivo* [34]. Our results for the first time demonstrate that deficiency of IGF-1R paradoxically confers resistance to oxidative stress induced apoptosis in C2C12 myoblasts. The exact mechanism of increased Akt activation in C2C12 myoblasts lacking IGF-1R remains to be determined.

## Supporting Information

Figure S1 **H_2_O_2_ does not affect IGF-1 induced Akt phosphorylation.** (A) C2C12 myoblasts were reverse-transfected in 6-well plates with 10 nM negative control siRNA (si-Con) and 10 nM siRNA against IGF1R (KD). Medium was changed after 24 h, and cells were maintained for another 24 h (90–95% confluent). After 48 h of initial transfection, myoblasts were serum starved for 2 h (0.1% BSA in DMEM) treated with rhIGF-1 (125 ng/ml) for 30 min (lane 3 and 4), H_2_O_2_ (400 µM) for 30 min (lane 5 and 6) or pre treatment with rhIGF-1 (125 ng/ml) for 30 min followed by H_2_O_2_ (400 µM) treatment for 30 min (lane 7 and 8). (B) Cells were grown and treated in similar way, as mentioned in (A); except cells were pretreated with H_2_O_2_ (400 µM) for 30 min followed by rhIGF-1 (125 ng/ml) treatment for 30 min (lane 7 and 8). Then cells were harvested for protein lysates and subjected to Western blot analysis. Two independent experiments were done, and a representative blot is shown.
(TIF)

Figure S2 **H_2_O_2_ induced Akt phosphorylation is PI3 kinase dependent.** (A) C2C12 myoblasts were reverse-transfected in 6-well plates with 10 nM negative control siRNA (si-Con) and 10 nM siRNA against IGF1R (KD). Medium was changed after 24 h, and cells were maintained for another 24 h (90–95% confluent). After 48 h of initial transfection, myoblasts were serum starved for 2 h (0.1% BSA in DMEM). Cells were treated with rhIGF-1 (125 ng/ml) for 30 min (lane 3 and 4), H_2_O_2_ (400 µM) for 30 min (lane 5 and 6), or pretreatment with wortmannin (Sigma cat.# 95455) followed by rhIGF-1 treatment for 30 min (lane 7 and 8) or H_2_O_2_ (lane 9 and 10) treatment for 30 min. (B) Cells were reverse transfected and maintained as described above. Cells were treated with H_2_O_2_ (400 µM) for 4-hours without (lane 1 and lane 2) or with (lane 3 and 4) wortmannin pretreatment. Cells were harvested for protein lysates and subjected to Western blot analysis. Blots are representative of two independent experiments.
(TIF)
