# Supplementary figures and images for: Deficiency of Insulin-Like Growth Factor-1 Receptor Confers Resistance to Oxidative Stress in C2C12 Myoblasts
Source: PLoS One. 2013 May 10;8(5):e63838. doi: 10.1371/journal.pone.0063838 (PMC3651254; doi:10.1371/journal.pone.0063838)

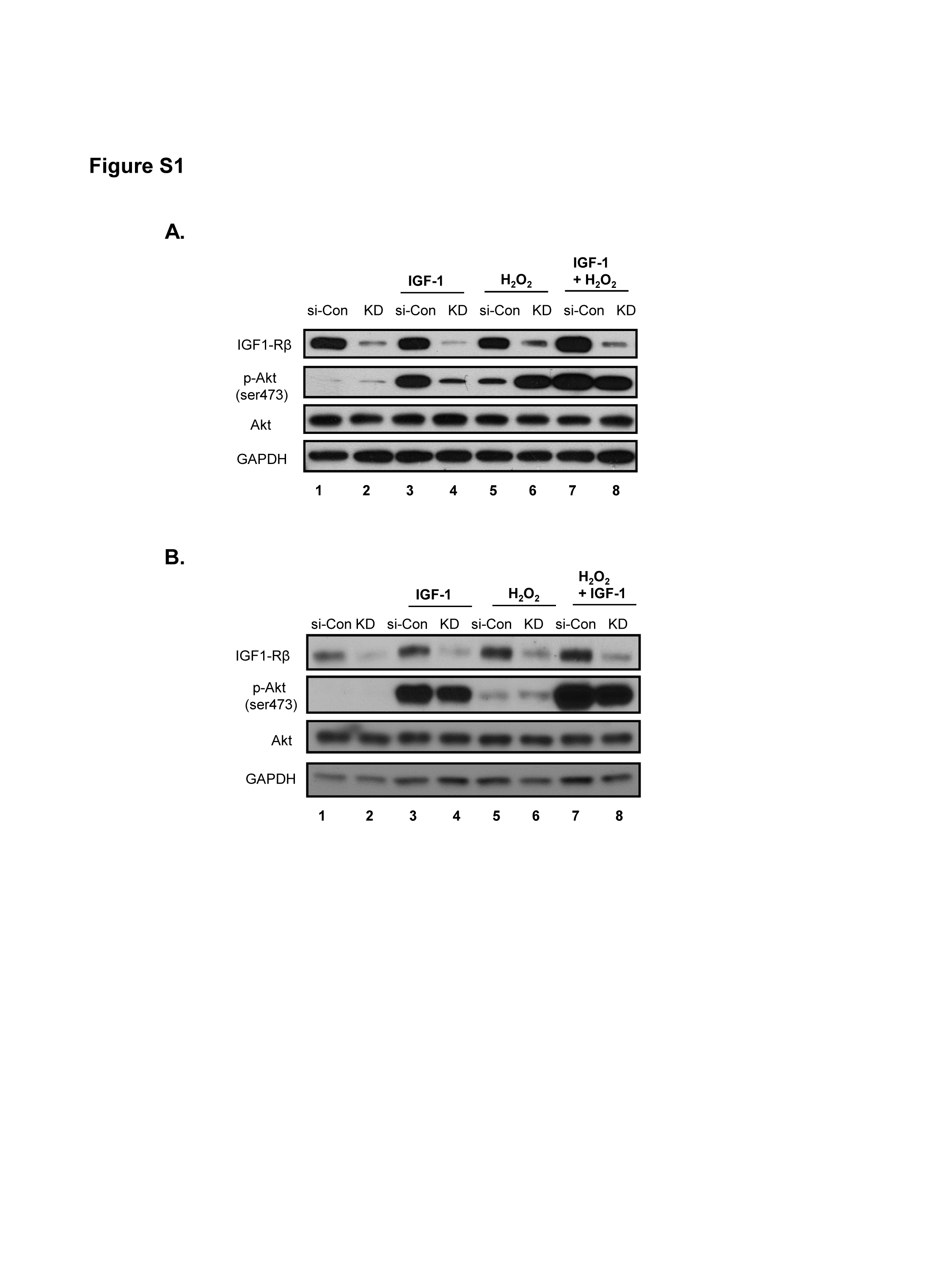

Supplement: Figure S1 — H2O2 does not affect IGF-1 induced Akt phosphorylation. (A) C2C12 myoblasts were reverse-transfected in 6-well plates with 10 nM negative control siRNA (si-Con) and 10 nM siRNA against IGF1R (KD). Medium was changed after 24 h, and cells were maintained for another 24 h (90–95% confluent). After 48 h of initial transfection, myoblasts were serum starved for 2 h (0.1% BSA in DMEM) treated with rhIGF-1 (125 ng/ml) for 30 min (lane 3 and 4), H2O2 (400 µM) for 30 min (lane 5 and 6) or pre treatment with rhIGF-1 (125 ng/ml) for 30 min followed by H2O2 (400 µM) treatment for 30 min (lane 7 and 8). (B) Cells were grown and treated in similar way, as mentioned in (A); except cells were pretreated with H2O2 (400 µM) for 30 min followed by rhIGF-1 (125 ng/ml) treatment for 30 min (lane 7 and 8). Then cells were harvested for protein lysates and subjected to Western blot analysis. Two independent experiments were done, and a representative blot is shown. (TIF) [file pone.0063838.s001.tif]

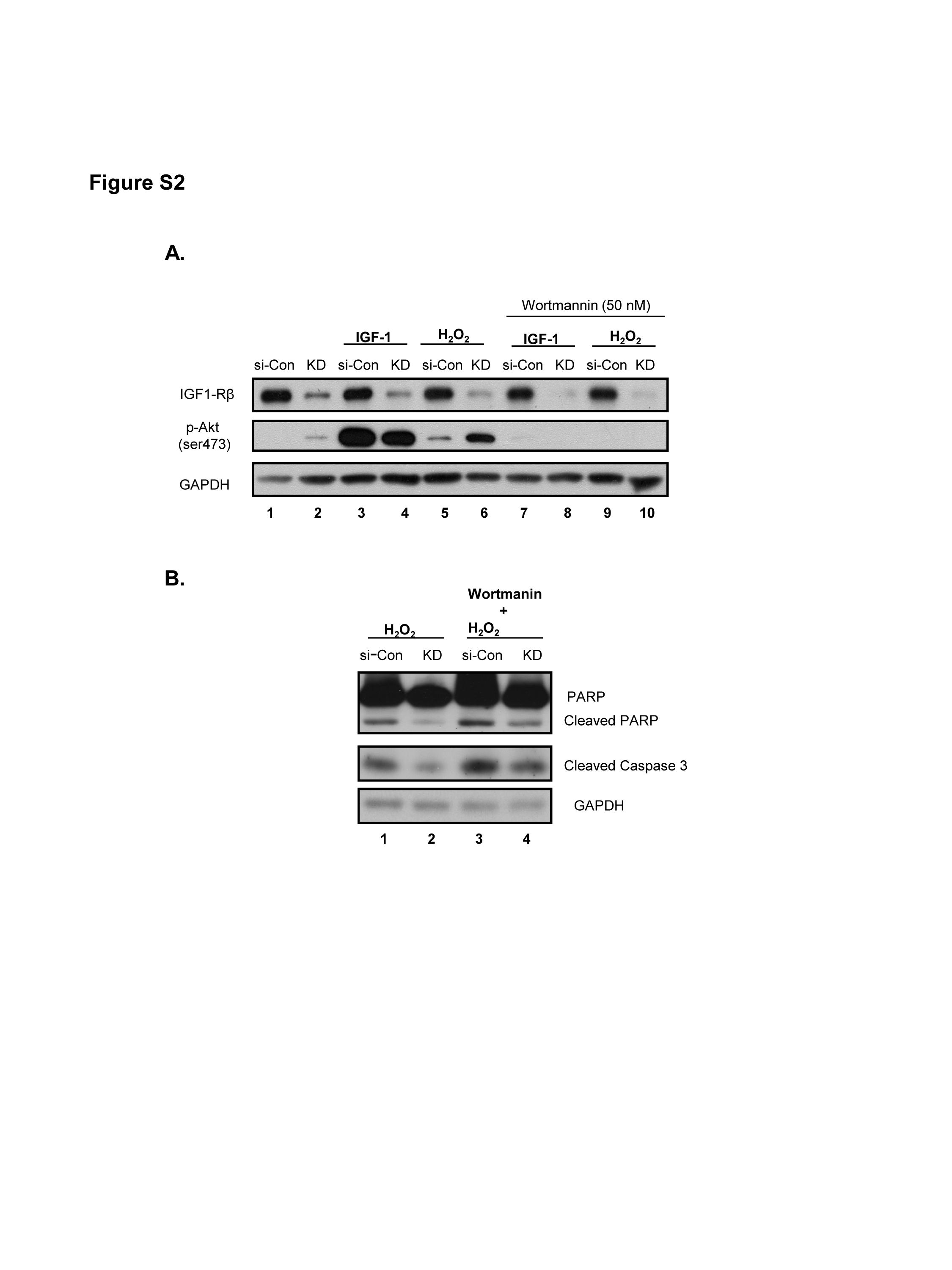

Supplement: Figure S2 — H2O2 induced Akt phosphorylation is PI3 kinase dependent. (A) C2C12 myoblasts were reverse-transfected in 6-well plates with 10 nM negative control siRNA (si-Con) and 10 nM siRNA against IGF1R (KD). Medium was changed after 24 h, and cells were maintained for another 24 h (90–95% confluent). After 48 h of initial transfection, myoblasts were serum starved for 2 h (0.1% BSA in DMEM). Cells were treated with rhIGF-1 (125 ng/ml) for 30 min (lane 3 and 4), H2O2 (400 µM) for 30 min (lane 5 and 6), or pretreatment with wortmannin (Sigma cat.# 95455) followed by rhIGF-1 treatment for 30 min (lane 7 and 8) or H2O2 (lane 9 and 10) treatment for 30 min. (B) Cells were reverse transfected and maintained as described above. Cells were treated with H2O2 (400 µM) for 4-hours without (lane 1 and lane 2) or with (lane 3 and 4) wortmannin pretreatment. Cells were harvested for protein lysates and subjected to Western blot analysis. Blots are representative of two independent experiments. (TIF) [file pone.0063838.s002.tif]
